# Editorial: Neuroimaging Approaches to the Study of Tinnitus and Hyperacusis

**DOI:** 10.3389/fnins.2021.700670

**Published:** 2021-07-14

**Authors:** Qian Chen, Han Lv, Yu-Chen Chen, Jae-Jin Song, Zhenchang Wang

**Affiliations:** ^1^Department of Radiology, Beijing Friendship Hospital, Capital Medical University, Beijing, China; ^2^Department of Radiology, Nanjing First Hospital, Nanjing Medical University, Nanjing, China; ^3^Department of Otorhinolaryngology-Head and Neck Surgery, Seoul National University Bundang Hospital, Seongnam-si, South Korea

**Keywords:** tinnitus and hyperacusis, structural MRI, resting-state fMRI, ASL, EEG, multimodality, sound therapy, brain reorganization

Approximately 12% of adults experience tinnitus, but about 1% experience severe tinnitus that is in some cases disabling. Tinnitus is often accompanied by hyperacusis, a debilitating condition in which moderate-intensity sounds are perceived as extremely loud. To date, a lot of evidence has supported that tinnitus and hyperacusis is a problem of the central nervous system that is caused or triggered by peripheral hearing loss (HL). Recent neuroimaging studies, such as structural magnetic resonance imaging (including diffusion tensor/spectrum imaging, DTI/DSI), resting-state functional magnetic resonance imaging (rs-fMRI), arterial spin labeling (ASL), and quantitative electroencephalography (EEG), have revealed significant structural and functional alterations of the brain associated with tinnitus and hyperacusis. From these studies, many key concepts linking tinnitus and/or hyperacusis to enhanced central gain, altered functional connectivity (FC) in neural networks, aberrant rhythmic activity in cortical networks, spontaneous hyperactivity, and aberrant activity in regions associated with anxiety, attention, emotion, and memory have emerged. In this Research Topic for Frontiers in Neuroscience, we bring together a collection of work from experts in the field that both summarizes past findings and introduces current advances to our understanding of non-pulsatile tinnitus and HL mechanisms using neuroimaging approaches. Hereinafter, we provide a summary of their contributions. The clinical and demographic data of the participants from all studies in the Research Topic are presented in Supplementary Table 1.

## Structural MRI Approaches

Tinnitus and hyperacusis can lead to significant brain structural changes that are closely related to patients' dysfunction. Structural MRI (including T1-weighted imaging and DTI) would be a useful measurement to evaluate these changes. In their article, using graph-theory-based analysis, Lin et al. provided the first morphological evidence of altered topological patterns of the brain networks in tinnitus patients. Moreover, they considered that the heightened efficiency of the brain network and altered auditory-limbic connection of patients would be compensation for the auditory deafferentation. Besides, Chen, Wang et al. explored the possible reorganization of brain white matter (WM) in tinnitus patients and found that tinnitus can cause significant brain WM microstructural alterations, and most of them were not auditory-related. More interestingly, these changes may be irrespective of the duration of tinnitus perception or other clinical performances. In the research of Zhang et al. they applied fiber tracking analysis and reported that sudden HL patients exhibited altered WM integrity in the auditory neural pathway that is associated with disease severity. Structural MRI can also be used for the evaluation of therapeutic outcomes. For example, Wei et al. investigated the changes in gray matter volume before and after narrow-band sound stimulation and found that sound therapy had a normalizing effect on the gray matter atrophy caused by tinnitus.

## Resting-State FMRI Approaches

As a powerful technique for characterizing the intrinsic brain activity and functional- or even network connectivity, rs-fMRI can provide useful information for us to better understand the neural mechanism of tinnitus and hyperacusis. For example, in the study of Cai et al. using amplitude of low-frequency fluctuation and seed-based FC, they found that abnormal alterations of regional activity in the central auditory system (such as the higher-order auditory cortex and inferior colliculus) existed. Moreover, these changes could result in increased FC among the auditory network, cerebellum, and limbic system, which could be another possible mechanism of tinnitus generation. The frontostriatal circuit plays a very important role in evaluating and modulating tinnitus perception signals. Using granger causality analysis, Xu J-J. et al. confirmed the neural basis of the frontostriatal gating control of tinnitus sensation and its contribution to tinnitus distress.

Tinnitus patients almost always have different degrees of hearing loss or hearing impairment; thus, we need to explore the possible neural mechanism of tinnitus with HL or even HL itself. In this regard, Zhou et al. explored the intraregional brain activity and FC in acute tinnitus patients with HL. Their study provided evidence that tinnitus with HL showed abnormal intraregional neural activity and disrupted connectivity in the hub regions of some non-auditory networks in the early stage, such as the default mode network (DMN), attention network, visual network, and executive control network. Meanwhile, Cai, Xie et al. reported aberrant functional and causal connectivities in both the auditory and non-auditory cortices in acute tinnitus patients with HL.

In addition, Xie et al. even discussed the neural mechanism in patients with hearing impairment, they found that there were significant differences in intrinsic brain activities and FC in unilateral hearing impairment patients, and the disease severity was associated with the FC values in some auditory or limbic-related regions. Moreover, Xu X-M. et al. found that patients with long-term HL would show disrupted spatial and temporal brain connectivity in the salience network, which is closely associated with hearing impairment-induced neuropsychiatric symptoms (such as cognitive impairments, depression, and anxiety).

Rs-fMRI can also be used to predict and evaluate the therapeutic efficacy of tinnitus. For example, Han L. et al. presented evidence that tinnitus patients' baseline FC characteristics can be used to predict the outcomes of sound therapy (narrow band noise). More importantly, they supposed that the connectivity of the thalamus at baseline would be a more reliable and objective neuroimaging-based indicator for effectiveness predicting of the therapy. Meanwhile, Zimmerman et al. applied resting-state FC analysis to evaluate the outcome of mindfulness-based cognitive therapy and found that the changes of amygdala-parietal connectivity would be a brain imaging marker of successful treatment. In short, rs-fMRI (including regional brain activity, undirected or directional connectivity, and network) can be a useful method to characterize brain functional alterations that may contribute to the occurrence or development of tinnitus and to predict or even evaluate patients' therapeutic outcomes.

## ASL Approach

Currently, few studies are using ASL to investigate the blood flow or neural mechanism of tinnitus and hyperacusis. In a recent study, the researchers found that tinnitus patients showed abnormal blood flow in the auditory cortex and the DMN (Xia et al.). More importantly, they considered the patients may benefit from blood glucose control in terms of tinnitus-related emotional dysfunction, such as cognitive function, distress, and depression.

## EEG Approaches

EEG can be used to characterize the brain reorganization features that may contribute to tinnitus and hyperacusis. For instance, using EEG analysis, both Lee, Choi et al. and Asadpour et al. discussed the possible neural mechanism of tinnitus. Asadpour et al. proved that tinnitus may be the result of the aberrant prediction error caused by the abnormal frequency of the stimuli, while Lee, Choi et al. reported that as our brain works in a Bayesian manner, tinnitus develops only if the deafferented brain updates the missing auditory information and the pregenual anterior cingulate cortex (pgACC)-based top-down gatekeeper system is actively involved and this process is also associated with the DMN. Meanwhile, based on resting-state EEG analysis (microstates and FC analysis), Cai, Chen et al. found that sudden HL patients not only showed alterations of central neural networks but also inhibition of brain area activity and change in FC. Moreover, EEG-related analysis can also be used to predict the efficacy of tinnitus pre-treatment and evaluate the prognosis after treatment. In this regard, Han J. et al. demonstrated that the ongoing cortical oscillatory activity before the hearing aids treatment may predict symptom improvement of patients with HL and tinnitus. Besides, in their research, Lee, Rhee et al. provided us the possible reason why tinnitus retraining therapy (TRT) is efficacious in most tinnitus patients: TRT induced habituation via modulation of FCs between the auditory system and the limbic and autonomic nervous systems, which may improve tinnitus-related distress. Additionally, Wang et al. explored the possible neural mechanism of hearing recovery after cochlear implantation in patients with HL by EEG analysis, and they suggested that the changes of some EEG features reflect the process of auditory function remodeling and may be a predictor for effect of cochlear implantation.

## Multimodality Imaging Approaches

A combination of the methods mentioned above would provide us more useful information about the underlying neural mechanism of brain reorganization tinnitus and hyperacusis. In a study performed by Luan et al. they conducted a combination of structural MRI, rs-fMRI, and DTI and demonstrated that the dorsolateral prefrontal cortex played an important role in patients with HL, as it recruited the auditory area into cross-sensory and higher-order processing through a top-down control. They provided evidence about the mechanism of cross-modal reorganization and cognitive participation in HL, whether with tinnitus or not. Moreover, Tang et al. applied multimodal imaging analysis as well, they reported the consistency of functional and structural pathways in the amygdala in HL patients. More importantly, they proved that the amygdala connectivity changes may be a potential mechanism underlying the HL-related emotional impairments. Besides, in a recent study conducted by Chen, Lv et al. it was demonstrated that sound therapy (narrow-band sound noise) has a significant effect on brain structural and network-level reorganization in idiopathic patients without HL. Additionally, in Hu et al. mini-review, they reviewed fMRI studies in a broader sense (including rs-fMRI, DTI, ASL, and so on) published in recent years on the neuroimaging mechanisms of tinnitus. The results have revealed various neural network alterations in tinnitus patients (such as vision network), which is confirmed by the study of Li et al. as they proved that the pre-response level of individuals with tinnitus showed a loss in vision dominance. They speculated the abnormality may be due to the reduced interference of visual information in auditory processing. These methods can also be applied for further researches on neural mechanisms of hyperacusis.

## Conclusion

These articles highlighted avenues for the research of tinnitus. New methods that can predict therapeutic outcomes before treatment and that can objectively evaluate treatment effects after therapy are provided. As can be appreciated, the articles covered a wide range of advances and new insights in our understanding of the neural mechanism underlying tinnitus and/or HL.

## Author Contributions

QC was responsible for drafting of the manuscript, final approval of the version of the manuscript to be published, and agreement to be accountable for all aspects of the work. ZW and HL was responsible for manuscript revision, final approval of the version of the manuscript to be published, and agreement to be accountable for all aspects of the work. Y-CC and J-JS was responsible for final approval of the version of the manuscript to be published and agreement to be accountable for all aspects of the work. All authors contributed to the article and approved the submitted version.

## Conflict of Interest

The authors declare that the research was conducted in the absence of any commercial or financial relationships that could be construed as a potential conflict of interest.

