# Supplementary material for: Editorial: Neuroimaging Approaches to the Study of Tinnitus and Hyperacusis
Source: Front Neurosci. 2021 Jul 14;15:700670. doi: 10.3389/fnins.2021.700670 (PMC8316917; doi:10.3389/fnins.2021.700670)
Supplement: Supplementary file 1 [file Table_1.DOCX]

**Supplementary table 1. List of all studies included in the research topic: subjects’ demographic and clinical characteristics (Ranking by first author's surname).**

| study | Modality/  Method of  analysis | Male: Female | | Mean age ± SD | | | Reported contrasts | Scanner | Processing software | Smoothing kernel (mm) | Statistical threshold | | MNI or Tal |
| --- | --- | --- | --- | --- | --- | --- | --- | --- | --- | --- | --- | --- | --- |
|  |  | Patients | Control | patients | Control | |  |  |  |  |  |  |  |
| Asadpour A et al. (2020) | EEG | 22:6 | 8:3 | 42.00 ±5.00 | 29.00 ±8.00 | | TIC > HC | NA | EEGLAB | NA | p<0.001, uncorrected | | NA |
| Cai W et al., (2019) | Rs-fMRI | 6:10 | 5:10 | 35.33±10.70 | 35.00±10.10 | | HC > TIN TIC > HC | GE 3.0T | DPABI | 8 | P<0.05, FWE corrected | | MNI |
| Cai Y et al. (2019a) | EEG | 9:16 | 10:17 | 46.16 ±13.15 | 41.48±13.53 | | HC > TIN | NA | EEGLAB13 | NA | p < 0.05,  uncorrected | | NA |
| Cai Y et al. (2019b) | EEG | 9:16 | 10:17 | 46.16 ±13.15 | 41.48±13.53 | | HC > TIN | NA | EEGLAB13 | NA | P<0.01, SnPM corrected | | MNI |
| Cai Y et al. (2020) | Rs-fMRI | 10:14 | 12:9 | 43.57±15.42 | 44.48±17.05 | | HC > TIN TIC > HC | Philips 3.0T | SPM12 | 4 | P<0.05, FWE corrected | | MNI |
| Chen Q et al. (2020) | DTI | 8:12 | 10:12 | 39.70±12.53 | 43.7±10.47 | | HC > TIN TIC > HC | Siemens 3.0T | FSL | 6 | P<0.05, FWE corrected | | MNI |
| Chen Q et al. (2021) | Multimodality | 6:7 | 9:9 | 42.23±13.9 | | 45.33±9.64 | HC > TIN TIC > HC | Siemens 3.0T | SPM12 | 6 | P<0.05, FDR/FWE corrected | | MNI |
| Han JJ et al. (2020) | EEG | 14:19 | NA | 64.60 ± 14.60 | NA | | NA | NA | LORETA-KEY | NA | P<0.05, SnPM corrected | | MNI |
| Han L et al. (2019) | Rs-fMRI | 12:15 | 12:15 | 37.50 ± 10.60 | 38.4 ± 11.3 | | TIC > HC | GE 3.0T | SPM8 | 6 | P<0.05, FDR corrected | | MNI |
| Hu J et al. (2021) | Multimodality | NA | NA | NA | | NA | NA | NA | NA | NA | NA | NA | |
| Lee S et al. (2020) | EEG | 6:7 | NA | 30-81/30-74 | NA | | Increase in TIC | NA | sLORETA | NA | P<0.05, SnPM corrected | | MNI |
| Lee S et al. (2019) | EEG | 20:17 | NA | 55.00 ±11.90 | NA | | NA | NA | LORETA-KEY | NA | P<0.05, SnPM corrected | | MNI |
| Li Z et al. (2019) | Behavioral | 21:17 | 16:15 | 28.23±6.20 | | 22.63±2.24 | HC > TIN | NA | NA | NA | p<0.05, uncorrected | NA | |
| Lin X et al. (2020) | SMRI | 31:15 | 26:20 | 40.90 ±12.50 | 42.3±14.2 | | TIC > HC | Philips 3.0T | GAT | 10 | P<0.05, FDR corrected | | MNI |
| Luan Y et al. (2019) | Multimodality | 21:14 | 17:18 | 54.49±9.45 | 55.97±7.80 | | HC > TIN TIC > HC | Siemens 3.0T | SPM8/FSL | 6 | P<0.05, FDR corrected | | MNI |
| Tang T et al. (2020) | Multimodality | 23:15 | 18:19 | 54.11±9.25 | 52.51±9.25 | | HC > TIN TIC > HC | Siemens 3.0T | SPM12 | 6 | P<0.05, FDR corrected | | MNI |
| Wang S et al. (2021) | EEG | 9:6 | 5:5 | 19-52 | 22-30 | | HC > TIN TIC > HC | NA | EEGLAB12 | NA | p<0.005, uncorrected | | NA |
| Wei X et al. (2020) | SMRI | 12:15 | 12:15 | 46.60 ± 9.90 | 46.40±12.00 | | HC > TIN | GE 3.0T | SPM8 | 6 | P<0.05, FDR corrected | | MNI |
| Xia W et al. (2021) | ASL | 21:37 | 16:23 | 49:81 ± 10:57 | 47:46±11:69 | | HC > TIN | Philips 3.0T | SPM12 | 6 | P<0.05, FWE corrected | | MNI |
| Xie X et al. (2019) | Rs-fMRI | 38:29 | 18:14 | 45.82±11.00 | 44.45±10.39 | | TIC > HC | GE 3.0T | SPM12 | 4 | p < 0.001,  uncorrected | | MNI |
| Xu J et al. (2019) | Rs-fMRI | 18:32 | 21:34 | 50:20±11:19 | 46:82±11:99 | | HC > TIN TIC > HC | Philips 3.0T | DPABI | 6 | p<0.001, GRF corrected | | MNI |
| Xu X et al. (2019) | Rs-fMRI | 22:10 | 14:16 | 54.50±9.30 | 53.60±8.00 | | HC > TIN | Siemens 3.0T | SPM12 | 6 | p < 0.001, GRF corrected | | MNI |
| Zhang Z et al. (2020) | DTI | 29:31 | 13:12 | 50.70±11.80 | 45.20±13.20 | | HC > TIN | Siemens 3.0T | DSI Studio | NA | p < 0.002,  uncorrected | | MNI |
| Zhou G et al. (2019) | Rs-fMRI | 14:14 | 12:19 | 41.20±11.61 | 45.40±14.32 | | HC > TIN TIC > HC | Philips 3.0T | SPM8 | 4 | p<0.001, AlphaSim corrected | | MNI |
| Zimmerman B et al. (2019) | Rs-fMRI | 5:7 | NA | 51.42 ± 10.63 | NA | | Decrease in TIC | Siemens 3.0T | SPM12 | 8 | P<0.05, FWE corrected | | MNI |

Note: TIN = tinnitus; HC = healthy control; MNI = Montreal Neurological Institute; NA = not available; SPM = statistical parametric mapping; FDR = false discovery rate; FWE = family-wise error; GFR = Gaussian Random Fields; DPABI = Data Processing & Analysis for Brain Imaging; EEG = electroencephalography; DTI = diffusion tensor imaging; DSI = Diffusion spectrum imaging; SMRI = structural magnetic resonance imaging; Rs-fMRI = resting-state functional magnetic resonance imaging.
